# Familial hepatocellular carcinoma: ‘A model for studying preventive and therapeutic measures’

**DOI:** 10.1016/j.amsu.2018.09.035

**Published:** 2018-09-28

**Authors:** Elroy Patrick Weledji

**Affiliations:** Department of Surgery, Faculty of Health Sciences, University of Buea, PO Box 126, Limbe, S.W. Region, Cameroon

**Keywords:** Hepatocellular, Carcinoma, Familial, Hepatitis B, Prevention, Prognosis

## Abstract

Hepatocellular carcinoma (HCC) is the fifth most common cancer worldwide, with more than 80% of cases found in endemic areas of hepatitis B such as Africa or East Asia. A family history of liver cancer increases HCC risk, independently of hepatitis. The combination of family history of liver cancer and hepatitis B/C serum markers is associated with an over 70-fold elevated HCC risk and poor prognosis. Only limited attention has been given to the role of primary genetic factors in HCC, but scattered anecdotal reports have identified familial aggregations of HCC. This article reviewed the literature on familial hepatocellular carcinoma and suggest that familial HCC may be a good model for studying preventive and therapeutic measures.

## Highlights

• Highest risk for HCC occur when a hereditary component acts in concert with hepatitis B virus.
• Different single nucleotide polymorphisms affect various biological pathways predisposing other risk factors.
• More extensive investigation of the genetic hypothesis of HCC and its fibrolamellar variant required.
• Prevention with neonatal vaccination is better than cure.

## 1 Introduction

HCC is one of the most commonly diagnosed cancer in the world with the incidence of 5.6% of total cancer cases [1,2]. Hepatocellular carcinoma (HCC) usually affects patients aged 50–70 years but earlier onset (25–40 years) may occur in hepatitis B endemic areas [3]. 70–90% of HCC develop on a background of cirrhosis but the hepatitis B virus is directly oncogenic and can cause HCC in the absence of cirrhosis [[3], [4], [5], [6]]. Limited attention has been given to the role of primary genetic factors in HCC especially as familial clusters of HCC are mostly described in areas with endemic HBV infection (Table 1). Growing evidence suggests that a family history of liver cancer significantly increases HCC risk with an aggressive nature. The hereditary component may act in concert with environmental factors such as hepatitis B [[7], [8], [9], [10]]. A multifactorial inheritance including novel DICER I germline mutation and altered liver zonation could contribute to the risk, regardless of viral hepatitis infection [9]. Different single nucleotide polymorphisms may increase the risk of HCC and the effect on various biological pathways may predispose the manifestations of other risk factors such as aflatoxin (from fermented crops). A classical Mendelian inheritance of HCC is limited to rare cases including the rare monogenic diseases such as haemochromatosis, tyrosinaemia type 1 and alpha 1 antitrypsin deficiency [7,10].

**Table 1:**
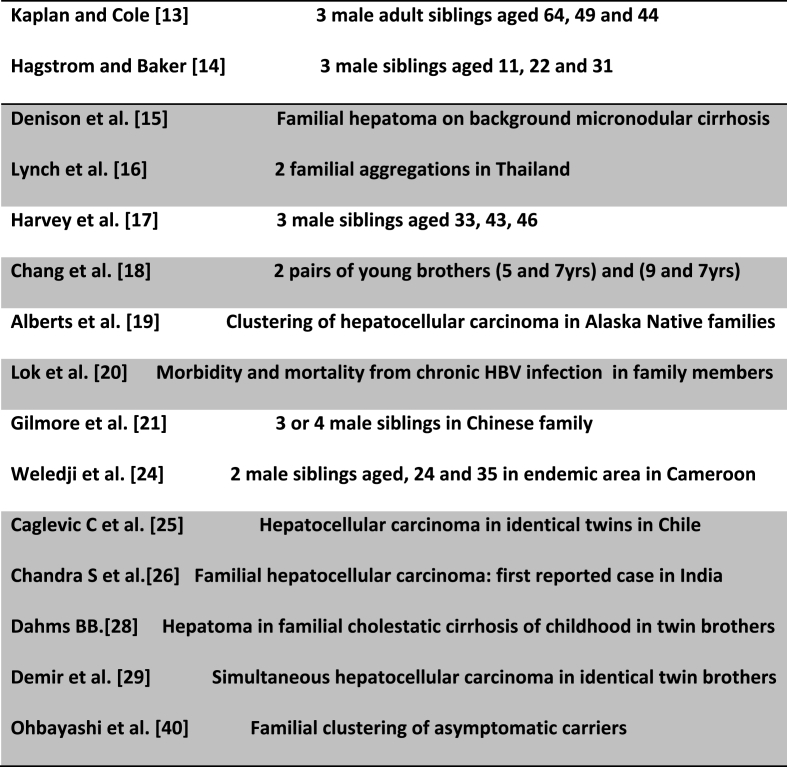
Familial hepatocellular carcinoma.

## 2 Discussion

### 2.1 Familial hepatocellular carcinoma

**Fig. 1:**
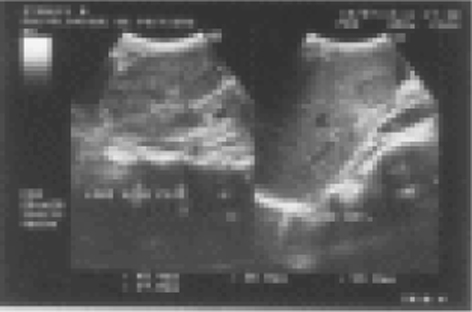
Transabdominal ultrasonography of liver showing multinodular HCC in left lobe and a nodular HCC with posterior enhancement in right lobe. (with permission) [19].

In endemic areas where HCC is common, HBV infection is considered to be important risk factor for familial HCC. However, studies from the western world concluded that a family history of HCC increases the risk of familial HCC independently of hepatitis [11,12], but, hepatitis B/C serum markers is associated with an over 70- fold elevated HCC risk [11]. As HBV can be directly oncogenic, the highest risk for HCC may occur in families in which a hereditary component is acting in concert with hepatitis B virus. Lui et al. [3] concluded in their study that familial HCC has earlier age of onset with associated aggressiveness. Familial occurrence of HCC was first reported by Kaplan and Cole which involved 3 male adult siblings aged 64, 49 and 44 [13]. Hagstrom and Baker [14] described HCC in 3 male siblings aged 11, 22 and 31 with no evidence of associated hepatic disease. Denison et al. [15] reported familial hepatoma on a background micronodular cirrhosis associated with hepatitis-associated antigen. Lynch et al. [16] reported two familial aggregations of HCC from Thailand. The maternal transmission of HBsAg and susceptibility to HCC is suggested by the pattern of involvement, with the affected mother and three sons (one of a different father) reported by Harvey et al. [17]. Chang et al. [18] reported fraternal hepatocellular carcinoma in young children in two families. Alberts et al. [19) reported clustering of hepatocellular carcinoma in Alaska native families. Lok et al. [20] revealed the morbidity and mortality from chronic hepatitis B virus infection in family members of patients with malignant and non-malignant hepatitis B virus-related chronic liver disease. Gilmore et al. [21] observed familial clustering of HCC in a Chinese family and concluded that HCC observed in 3 or 4 male siblings even in the presence of hepatitis B infection seemed statistically unlikely to occur by chance. It was therefore suggested that another risk factor such as environmental and/or genetic factors may be involved [22,23]. Weledji et al. [24] reported a black African family in which the clinical diagnosis of familial HCC was made on two male siblings (aged 24 and 35) in Cameroon, W/Africa. The diagnosis of HCC was clinically-based on fever, weight loss, right hypochondrial pain from nodular liver lesions and refractory ascites. There was no underlying cirrhosis nor stigmata of chronic liver disease (Fig. 1). Even though the serology was negative, it was very likely that the brothers were carriers of HBsAg being in the endemic area. In these cases hepatitis B virus DNA (HBVDNA) and hepatitis B core antibody (HBcAb) tests although not available were mandatory. The fact that both siblings had same mother may suggest the possibility of vertical transmission of the hepatitis B surface antigen [17]. Incidentally, the father died about 3 years later from an unknown cause as an autopsy was not done. More recently, Caglevic et al. [25] reported HCC in identical twins in Chile. In 2016, there was the first reported case of familial hepatocellular carcinoma in 2 brothers aged 45 and 38 years in India who succumbed to their illness within three months of diagnosis [26]. Both patients were non-reactive for hepatitis B or C virus and there was no associated alcoholism, obesity, diabetes or smoking suggesting its independent association with genetic factors. This was corroborated by the family history which revealed the death of their mother 20 years previously with similar complaints at around the same age (48yrs) and under similar circumstances. This may suggest an inheritable genetic cause for the development of familial HCC. However, there was evidence of mild to moderate steatosis in the ultrasound-guided fine needle aspiration cytology (FNAC) smear of the liver lesions. The observation of steatohepatitic morphology in HCC is commonly seen in non-alcoholic fatty liver disease and suggests significant association with metabolic risk factors [27]. Previously, Dahms [28] had reported familial cholestatic cirrhosis of childhood in twin brothers, and, Denir et al. [29] demonstrated simultaneous presentation of hepatocellular carcinoma in identical twin brothers. Thus, there may be a role for using epigenetic studies in monozygotic twins to improve the understanding of HCC. By avoiding the confounding influence of constitutive genetic backgrounds, age or cohort effects, epigenetic variation analysis in twins would identify the susceptibility loci that may be sensitive to modification by the environment [30].

### 2.2 Fibrolamellar variant

The fibrolamellar variant of HCC (FHCC) is due to a heterozygous deletion on chromosome 19 that encodes a functional chimeric protein (DNAJB1-PRKACA) and characterized by being less aggressive with a normal alpha-fetoprotein (AFP) tumour marker and a female predominance [31,32]. This is in contrast to conventional HCC which has a two-to four-fold male predominance and occurs mostly in hepatitis B endemic areas [32]. Being rare, familial FHCC clustering has never been reported [33,34]. Nevertheless, although FHCC mostly occurs in non-hepatitis B endemic areas, some cases have been reported in relatively endemic areas [35]. It is characterized by the lack of symptoms until the tumour is sizeable and is thus often advanced when diagnosed. Serum neurotensin, a new tumour marker for FHCC may discriminate it from HCC especially as a negative or normal value of alpha fetoprotein (AFP) tumour marker does not exclude an HCC [32].

### 2.3 Tumour markers

AFP tumour marker is elevated in only 50–60% of cases of HCC [36]. However, global gene expression profiling revealed a small set of genes, SPINK 1, a secretory trypsin inhibitor as a potential HCC marker [37]. The overexpression of the apolipoprotein family (ApoA1, ApoA2, ApoC3, ApoE) and serum amyloid A protein in comparative proteomic profiles of HCC cases family members have indicated that genetic factors may account for familial aggregation of HCC [38].

### 2.4 Genetic susceptibility

**Fig. 2:**
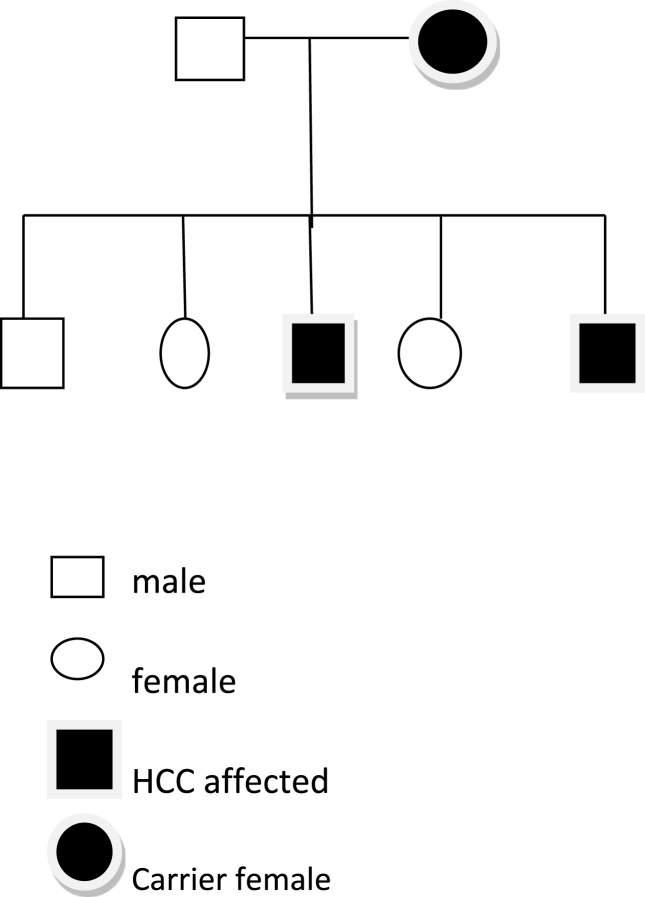
Family tree of two generations depicting carrier state commoner in males (with permission) [19].

There must be a genetic basis for aggressive liver malignancy occurring in siblings during their early adulthood [3,5,7,9,24]. The earlier onset of HCC and death in the younger sibling may harbour important implication for hereditary susceptibility to this lesion [3,24,39]. It may indicate a greater genetic contribution to persistent infection than in the older sibling. Up to 20% of the population in highly endemic areas may be asymptomatic HBsAg positive chronic carriers and occasionally infection without detectable serum HBsAg occurs [20,24,40]. In addition, changes in HBsAg variants in carrier children before and after universal vaccination have been reported [41]. Vertical transmission of the hepatitis B virus is probably a major reason for the high incidence of hepatitis B infection in endemic areas with infants acquiring the virus from infected mothers in utero, at birth or shortly after birth [17]. Genetic contribution may also explain the presence of healthy or non-healthy carriers of HBsAg, and the carrier state being commoner in males (Fig. 2) [[7], [8], [9],^20^,^41^,^42^]. HCC-prone families of the familial type discussed may provide a powerful model for studying preventive and therapeutic measures. It would seem prudent that hepatitis vaccination be given the highest priority to those individuals where the HCC yield is increased [43,44]. With the impaired immunity of the baby compared with later life the infant is at increased risk of developing persistent hepatitis B carriage with the sequelae of chronic active hepatitis, progressive liver damage and hepatocellular cancer [5]. The optimum timing for immunisation in conjunction with the administration of hepatitis B immunoglobulin at a contralateral site should be immediately after birth or within 12 h [45,46]. It is also important to screen all first degree relatives of patients with HCC in order to detect early and asymptomatic disease.

## 3 Conclusions

A greater attention should be given to the role of primary genetic factors in HCC and their interaction with environmental factors, such as HBV infection and aflatoxin exposure. A proper neonatal hepatitis B immunisation scheme would reduce the incidence of HCC in endemic areas. A positive family history is associated with earlier appearance and aggressiveness of HCC. Early surveillance and follow up of relatives for every case of HCC should provide early diagnosis and management of familial HCC. A more extensive investigation of the genetic hypothesis of HCC may help develop novel therapies that may improve the current poor prognosis of this disease.

## Provenance and peer review

Not commissioned, externally peer reviewed.

## Conflicts of interest

The author has no conflict of Interest.

## Sources of funding

None.

## Ethical approval

Ethical approval not required.

## Consent

Consent not required.

## Author contribution

EPW: Study design, drafting and writing of paper, literature search.

## Guarantor

The Dean, Prof M Ngowe Ngowe, Faculty of Health Sciences, University of Buea, PO Box 63, Buea, Cameroon.
